# Blocking of the IL-33/ST2 Signaling Axis by a Single-Chain Antibody Variable Fragment (scFv) Specific to IL-33 with a Defined Epitope

**DOI:** 10.3390/ijms21186953

**Published:** 2020-09-22

**Authors:** Soo Bin Park, Sun-Jick Kim, Sang Woo Cho, Cheol Yong Choi, Sangho Lee

**Affiliations:** Department of Biological Sciences, Sungkyunkwan University, Suwon 16419, Korea; stephanie7007@daum.net (S.B.P.); godmouse@skku.edu (S.-J.K.); aksgdlwkd@skku.edu (S.W.C.); choicy@skku.ac.kr (C.Y.C.)

**Keywords:** interleukin 33, ST2 receptor, scFv, C2_2E12

## Abstract

Interleukin 33 (IL-33) is an IL-1 family cytokine that plays a central role in immune system by regulating and initiating inflammatory responses. The binding of IL-33 to the suppressor of tumorigenicity 2 (ST2) receptor induces mitogen-activated protein kinases (MAPK) and nuclear factor κB (NF-κB) pathways, thereby leading to inflammatory cytokines production in type 2 helper T cells and type 2 innate lymphoid cells. To develop an antibody specific to IL-33 with a defined epitope, we characterized a single-chain antibody variable fragments (scFvs) clone specific to IL-33, C2_2E12, which was selected from a human synthetic library of scFvs using phage display. Affinity (*K*_d_) of C2_2E12 was determined to be 38 nM using enzyme-linked immunosorbent assay. C2_2E12 did not show cross-reactivity toward other interleukin cytokines, including closely related IL-1 family cytokines and unrelated proteins. Mutational scanning analysis revealed that the epitope of IL-33 consisted of residues 149–158 with key residues being L150 and K151 of IL-33. Structural modeling suggested that L150 and K151 residues are important for the interaction of IL-33 with C2_2E12, implicating that C2_2E12 could block the binding of ST2 to IL-33. Pull-down and in-cell assays supported that C2_2E12 can inhibit the IL-33/ST2 signaling axis. These results suggest that the scFv clone characterized here can function as a neutralizing antibody.

## 1. Introduction

Interleukin-1 (IL-1) family cytokines play important roles in regulating and initiating inflammatory and immunological responses [1]. The IL-1 family includes eleven cytokines comprising seven agonist ligands, three receptor antagonists, and an anti-inflammatory cytokine [2]. Interleukin 33 (hereafter called as “IL-33”) cytokine is identified as one of the IL-1 family agonist ligands [3]. It was first regarded as an alarmin that is released to signal immune system when a cell or tissue is damaged or stressed [4]. Recently, IL-33 has been considered as an important factor of the immune system involved in allergic inflammation and chronic diseases such as asthma, atopic dermatitis, and allergic rhinitis [5,6,7].

IL-33, expressed in endothelial cells, fibroblasts, epithelial cells, and other cells, binds to its receptor suppressor of tumorigenicity 2 (ST2)/interleukin 1 receptor-like 1 (IL1RL1), which formed heterodimer with co-receptor, IL-1 receptor accessory protein (IL1RAcP) [4,8]. There are two types of ST2 isoforms: the transmembrane form, ST2, and soluble form, sST2, covering residues 19 through 321 of the ectodomain (hereafter, we call all isoforms as simply “ST2”). The ST2 is expressed on various immune cells including innate lymphoid group 2 cells (ILC2s), mast cells, dendritic cells, macrophages, basophils, and type 2 helper T cells (T_h_2), and it is linked to T_h_2 effector functions [9,10]. IL-33 exerts its biological functions followed by binding to ST2 expressed in immune cells, and it is mainly associated with T_h_2 responses through the production of inflammatory cytokines IL-5 and IL-13 [3,11]. The heterodimer complex formation activates downstream signaling complex formation. Myeloid differentiation primary response 88 (MyD88) first binds to heterodimeric receptor and leads to the recruitment of interleukin-1 receptor-associated kinase 1 (IRAK1), IRAK4 and tumor necrosis factor (TNF) receptor-associated factor 6 (TRAF6), and these subsequently activate mitogen-activated protein kinases (MAPKs) and nuclear factor κB (NF-κB) signaling pathways to promote inflammatory cytokine production [9,12,13]. It seems that IL-33 has the potential to activate T_h_2 cytokine-mediated allergic inflammation and related diseases, suggesting that blockade of the IL-33/ST2 signaling axis can be a new therapeutic strategy for allergic inflammation and chronic inflammatory diseases [14,15,16].

Several strategies have been developed to suppress the IL-33 mediated downstream signaling pathway to prevent chronic diseases and allergic inflammation: antagonists against IL-33, and antagonists against ST2 or sST2 binding to IL-33 [17]. Here, we describe the discovery and characterization of single-chain variable fragment (scFv) monoclonal antibodies (mAbs) directly targeting IL-33 to inhibit IL-33 binding to ST2. Although there are diverse antibodies against IL-33 for various purposes, they are mainly derived from immunizing living organisms with immunoglobulin G forms [16,18] or monoclonal antibodies for IL-33 detection [15,19]. Since immunoglobulin G (IgG) forms of antibodies are hard to handle and not suitable to further engineering, we used a human synthetic single-chain variable fragment (scFv) antibody library to screen IL-33 specific mAbs in vitro.

The discovery of mAbs using phage display library was performed with five rounds of biopanning, and enzyme-linked immunosorbent assay (ELISA) was used to determine the antibodies affinity. Using immunoblotting, we observed their cross-reactivity, and two types of mutant-based epitope mapping were implemented to identify the binding epitope domains. The inhibition effect of antibody was verified by glutathione *S*-transferase (GST) pull-down assay and human cell-expressing ST2 and IL1RAcP-based assay. The antibody seems to have therapeutic function by interfering with IL-33 binding to the ST2 receptor, heterodimeric receptor complex formation, and blocking the IL-33/ST2 signaling axis.

## 2. Results

### 2.1. Selection of scFvs Specific to IL-33

Human IL-33 is composed of three domains: N-terminal nuclear domain (residues 1–65), central domain (residues 66–111) and C-terminal IL-1-like cytokine domain (residues 112–270) [3,8,20]. The N-terminal and central domains of IL-33 are cleaved by caspase-1 to produce the mature form [3]. IL-33 is susceptible to oxidation by forming disulfide bonds among cysteine residues (C208, C227, C232, and C259). IL-33 oxidation reportedly drives a conformational change and inactivates its ST2-dependent cytokine activities [21,22]. To prevent from oxidation, we mutated C208 and C232 to serine and compared the activity of C208S/C232S mutant with that of IL-33 wild-type (WT). The purity of WT and C208S/C232S mutant was confirmed by SDS-PAGE (Figure S1A,B). The recognition of IL-33 WT and C208S/C232S mutant by a selected scFv (see next section) was comparable as corroborated by SDS-PAGE and immunoblot analyses (Figure S1C). We used GST-IL-33 WT for biopanning and characterizations and IL-33 C208S/C232S mutant for cell signaling analysis.

We performed five rounds of biopanning to select scFvs specific to IL-33 using a large synthetic human scFv library in two distinct conditions according to the number of negative selections (Table S1). GST-IL-33 and GST were used as antigens for positive and negative selections of biopanning, respectively. Ten scFv clones with high OD_450_ values in response to IL-33 compared to the negative selection were selected by ELISA screening (Figure S2A). Of the ten clones, clones with mutations in the backbone frame or duplicated sequences were excluded through multiple protein sequence alignment (Figure S2B). Finally, six clones (C1_1E1, C2_1D5, C2_2A10, C2_2E1, C2_2E12, and C2_2H5) were chosen based on their high binding signals at 450 nm without mutations in the amino acid sequences. Multiple sequence alignment revealed that these six clones have different amino acid residues mostly in the third complementarity determining region in heavy chain (CDR-H3) and the second complementarity determining region in light chain (CDR-L2).

**Figure 1:**
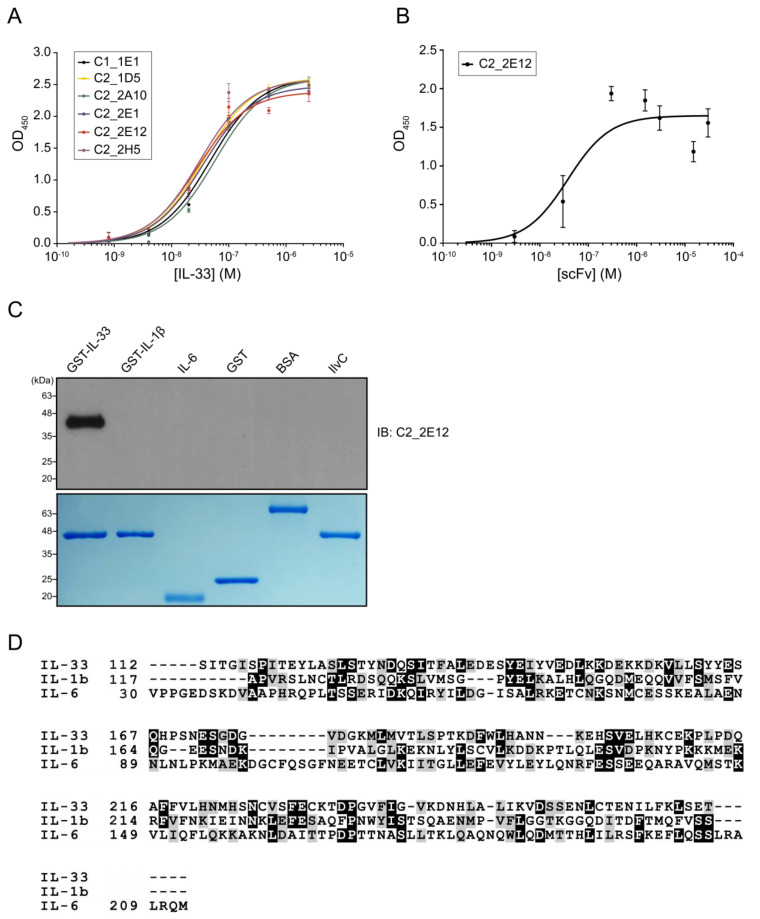
Molecular characterizations of anti-IL-33 single-chain antibody variable fragments (scFvs). Enzyme-linked immunosorbent assay (ELISA)-based affinity determination of anti-IL-33 scFvs. (**A**) Affinity determination of the top six clones that exhibited high binding signals against IL-33. (**B**) Affinity determination of the purified C2_2E12, which seems to have the highest binding signal and good protein condition among six clones. *K*_d_ values of C1_1E1, C2_1D5, C2_2A10, C2_2E1, C2_2E12, and C2_2H5 were estimated by kinetic analysis. ELISA was repeated three times. (**C**) Immunoblot analysis of the recombinant proteins using C2_2E12 for primary antibody (0.5 mg·mL^−1^, 1:100 dilution) and anti-hemagglutinin-horse radish peroxidase (anti-HA-HRP) for secondary antibody (0.2 mg·mL^−1^, 1:5000 dilution). Expression of recombinant interleukin cytokines (GST-IL-33, GST-IL1β, and GST-IL6) and unrelated proteins (GST, BSA, and IlvC) in *E. coli* BL21 (DE3) as revealed by SDS-PAGE analysis. IB, immunoblot. (**D**) Multiple protein sequence alignment of IL-33, IL1β, and IL6. BSA: bovine serum albumin, IL: interleukin.

The *E. coli* cell lysates containing overexpressed His_6_-tagged scFvs were prepared to determine the binding affinity of selected scFvs with IL-33. The dissociation constants (*K*_d_) values of C1_1E1, C2_1D5, C2_2A10, C2_2E1, C2_2E12, and C2_2H5 by ELISA were estimated to be 48, 36, 57, 35, 28, and 31 nM, respectively (Figure 1A). The *K*_d_ value of C2_2E12 that showed the highest affinity using cell lysate was further measured using the purified proteins by ELISA (Figure S2C and Figure S1B). The *K*_d_ value of the purified C2_2E12 was 38 nM (Figure 1B), which is consistent with the value estimated using the cell lysate. We selected C2_2E12 for further characterizations.

The cross-reactivity of C2_2E12 was checked for two interleukins belonging to the same subfamily (GST-IL-1β and IL-6) and three unrelated proteins (GST, bovine serum albumin (BSA) and IlvC). Immunoblot assay results showed that C2_2E12 only reacted with IL-33 (Figure 1C). It is interesting that C2_2E12 did not react with IL-1β and IL-6, since IL-33, IL-1β, and IL-6 belong to the same subfamily. Human IL-33 shows low sequence identities to IL-1β and IL-6 despite all three belonging to the same subfamily: 13.5% with IL-1β and 12.9% with IL-6, respectively (Figure 1D). The structure of IL-1β (PDB ID: 1L2H) is similar to that of IL-33 (PDB ID: 4KC3) with a root mean square deviation (r.m.s.d.) of 1.93 Å, while the structure of IL-6 (PDB ID: 1ALU) is completely different from that of IL-33. Given the low sequence similarities and structural differences, no cross-reactivity of C2_2E12 for IL-1β and IL-6 seems to be reasonable. The cross-reactivity results clearly demonstrate that C2_2E12 specifically binds to IL-33.

### 2.2. Epitope Mapping

**Figure 2:**
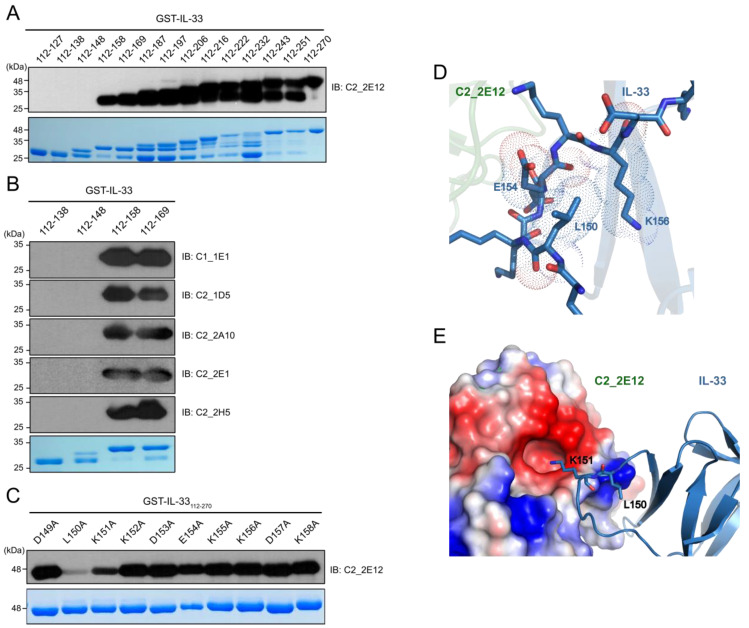
Epitope mapping of C2_2E12. (**A**) Immunoblot analysis of 14 GST-IL-33 deletion mutants to map the IL-33 epitope at secondary structural element level for scFv clone C2_2E12. Residue numbers of the mutants are shown. (**B**) Immunoblot analysis of four GST-IL-33 deletion mutants to map the IL-33 epitopes for scFv clones C1_1E1, C2_1D5, C2_2A10, C2_2E1, and C2_2H5. (**C**) Immunoblot analysis of alanine scanning for GST-IL-33_149_-_158_ for recognition by the scFv clone C2_2E12. Residue numbers and identities are shown. In panels (**A**) through (**C**), Coomassie Blue stained gel is shown at the bottom. Amount of the loaded protein per lane was 1 μg. The scFv clones were used as the primary antibody (0.5 mg·mL^−1^, 1:100 dilution), and anti-HA-HRP was used as the secondary antibody (0.2 mg·mL^−1^, 1:5000 dilution). (**D**,**E**) The HADDOCK-derived molecular docking of C2_2E12 (green) to IL-33 (PDB: 4KC3, blue) complex. A homology structural model for C2_2E12 was generated using SwissModel [24]. Both proteins are depicted as cartoon diagrams. (**D**) Residues of IL-33 in the epitope region recognized by C2_2E12 are represented as stick models. Dash lines represent van der Waals atomic distances in Å. (**E**) Electrostatic interactions between C2_2E12 and IL-33. The acidic pocket in C2_2E12 consists of N163, D164, S166, Y168, A218, and Y230. L150 and basic K151, the two key residues in the epitope region of IL-33 are depicted as stick models. The figures in the panels (**D**) and (**E**) were generated using PyMOL (Schrödinger).

To determine the epitope region in IL-33 for C2_2E12, a series of GST-IL-33_112_-_270_ N-terminal deletion mutants were constructed by the insertion of a stop codon at the end of each α-helix or β-strand of IL-33 based on the crystal structure of human IL-33 (PDB ID: 4KC3) [23] (Figure 2A). Immunoblot analysis revealed that residues 149–158 of the IL-33 comprised the epitope region, which corresponded to its receptor ST2 binding site in the crystal structure of the IL-33:ST2 complex (PDB ID: 4KC3). We found that the other five scFv clones (C1_1E1, C2_1D5, C2_2A10, C2_2E1, C2_2E12, and C2_2H5) also recognized the same epitope region in the IL-33 (Figure 2B). Alanine scanning mutagenesis was performed to determine the critical residue(s) in the epitope region (Figure 2C). Each residue in GST-IL-33_149-158_ was substituted to alanine by site-directed mutagenesis PCR. The effects of the IL-33 mutants were analyzed by immunoblots with C2_2E12 as the primary antibody. Alanine substitutions of L150 and K151 of IL-33 reduced the binding with C2_2E12, rendering these the key residues in the epitope region. To obtain further insights on alanine scanning results at the molecular level, we performed molecular docking between IL-33 and C2_2E12 using the HADDOCK server with restraints that only L150 and K151 residues of IL-33 and CDR residues of C2_2E12 should participate in interactions. Although alanine scanning data showed that L150 of IL-33 is a key residue of IL-33 and C2_2E12 binding, L150 seemed to not interact with any residue of C2_2E12. Alternatively, L150 seemed to possibly interact with the surrounding hydrophobic residues of the 149–158 epitope region of IL-33, and it also seemed to play an important role in maintaining the shape of the loop (Figure 2D). It seems that the L150A mutant inhibits the interaction between IL-33 and C2_2E12 by local conformational changes of the loop. The docked structural model of IL-33:C2_2E12 suggested that K151 of IL-33 seemed to interact electrostatically with the acidic pocket of C2_2E12 composed of D164, S166, Y168, A218, and Y230 (Figure 2E). This structural analysis with a docked model between IL-33 and C2_2E12 supports that L150 and K151 residues of IL-33 are important for their binding to C2_2E12.

### 2.3. Competitive Binding of C2_2E12 to IL-33:ST2 Complex

**Figure 3:**
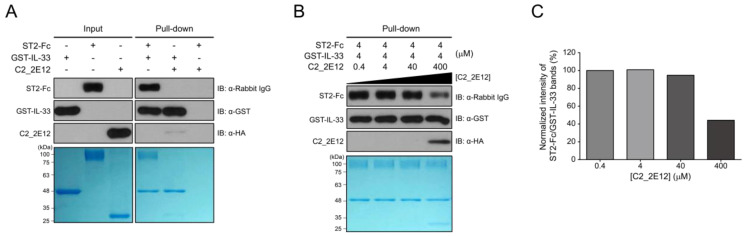
Interfering with IL-33 and suppressor of tumorigenicity 2 (ST2) complex formation by C2_2E12. GST pull-down assay was used to observe the interaction of C2_2E12 with IL-33 competitively with ST2 receptor in vitro. Pull-down assay was performed step by step. Immobilize GST-IL-33 in Glutathione Sepharose 4B and add ST2-Fc fusion proteins. Then, put C2_2E12 in a dose-dependent manner. Binding was performed for 30 min every step. Molar concentration of the proteins for binding were GST-IL-33: ST2-Fc: C2_2E12 = 1: 1: 0.1, 1:1:1, 1:1:10, and 1:1:100 (M). (**A**) Input loading (GST-IL-33 in lane 1, ST2-Fc in lane 2, and C2_2E12 in lane 3) and proteins binding test (lane 4, 5, and 6) with pull-down assay were visualized by immunoblot and SDS-PAGE. (**B**) Inhibition of IL-33 and ST2 binding by anti-IL-33 antibody in a dose-dependent manner. After 4 µM of GST-IL-33 was immobilized in resin and 4 µM of ST2-Fc was added to the resin, C2_2E12 (0.4, 4, 40, and 400 µM in lane 1, 2, 3, and 4) was added in a dose-dependent manner and visualized by immunoblot analysis and SDS-PAGE. (**C**) Quantification of the inhibitory effects of C2_2E12 for the IL-33:ST2 interaction. Band intensities of ST2-Fc in panel (**B**) were quantified using ImageJ and normalized by dividing them by those of GST-IL-33. Relative intensities in reference to that of ST2-Fc with 0.4 µM C2_2E12 are shown as a bar graph.

Residues 149–152 and 156 in the epitope of IL-33 for C2_2E12 are reportedly involved in the interaction with the ectodomain (residues 19–321) of ST2, which is present in both the transmembrane and soluble isoforms [23]. Since the epitope of IL-33 for C2_2E12 overlaps with the ST2 binding site, we hypothesized that the C2_2E12 could function as a blocking antibody in the IL-33/ST2 signaling axis. To test the hypothesis, in vitro GST pull-down assay was performed. The antibody fragment crystallizable (Fc) fusion of ST2, ST2-Fc, interacted with immobilized GST-IL-33 as expected. By contrast, ST2-Fc did not bind to the immobilized GST-IL-33 in the presence of C2_2E11 (Figure 3A). To investigate whether C2_2E12 inhibits IL-33:ST2 interaction in a dose-dependent manner, a series of concentrations of C2_2E12 were used for competitive binding assay GST pull-down assay. The IL-33:ST2 interaction was reduced at 100-fold molar excess of C2_2E12 (Figure 3B). Quantification of the IL-33:ST2 interaction in the presence of increasing concentrations of C2_2E12 showed that the IL-33:ST2 interaction decreased in a concentration-dependent manner, leading to about 40% level at 100-fold molar excess of C2_2E12 (Figure 3C). These results suggest that C2_2E12 can act as a neutralizing antibody in the IL-33/ST2 signaling axis in vitro.

### 2.4. C2_2E12 Can Neutralize IL-33/ST2 Axis Driving Downstream Signaling Pathway in Human Cell Line

**Figure 4:**
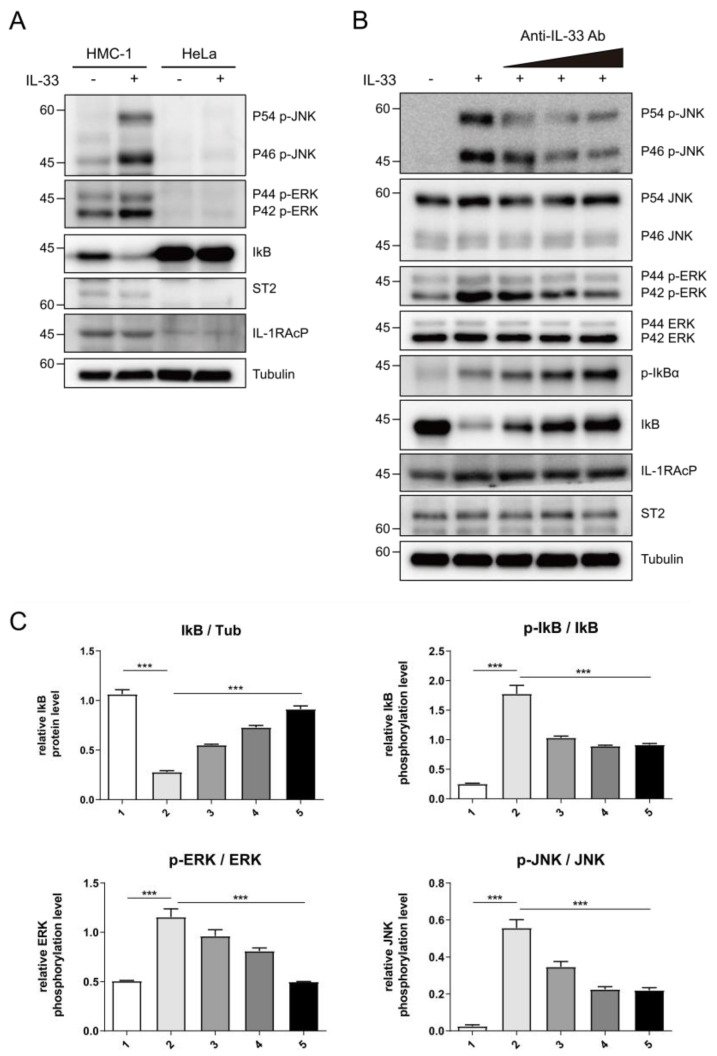
Intervention of IL-33/ST2 signaling axis by C2_2E12 antibody. (**A**) IL-33-mediated activation of nuclear factor κB (NF-κB) signaling in human mast cells (HMC-1) cells, but not in HeLa cells where ST2 and IL-1RAcP were not expressed. HMC-1 and HeLa cells were stimulated with mock or human IL-33 (1 ng·mL^−1^, 8 min), and cell lysates were analyzed by immunoblotting with the indicated antibodies. (**B**) The inhibition of IL-33-induced NF-κB signaling by C2_2E12 antibody in a dose-dependent manner. HMC-1 cells were treated with IL-33 alone or IL-33 pre-incubated with the increasing amounts of C2_2E12 antibody. Cell lysates were analyzed by immunoblotting with the indicated antibodies. IL-33 (1 ng·mL^−1^) was pre-incubated with increasing amounts of the C2_2E12 antibody (0.1, 0.5, and 2.0 ng·mL^−1^ in lanes 3, 4, and 5, respectively) for 15 min and treated to HMC-1 cells for 8 min. (**C**) Quantification of protein levels shown in (**B**). Band intensities in immunoblots were quantified using ImageJ. Data are expressed as the mean ± SEM. The statistical significance of differences was analyzed by one-way analysis of variance (ANOVA) followed by Bonferroni’s multiple comparison test (*** *p* < 0.001 compared to the indicated points; *n* = 3).

To corroborate the neutralizing effects of C2_2E12 in the IL-33/ST2 signaling axis in cells, we tested IL-33 induced MAPK and NF-κB pathways activation in human mast cells (HMC-1). HMC-1 cells expressed endogenous ST2 receptor and IL-1RAcP co-receptor, unlike HeLa cells (Figure 4A). The levels of phosphorylated extracellular signal-regulated kinase (ERK) and c-Jun N-terminal kinase (JNK) in the MAPK pathway were increased by the IL-33 C208S/C232S:ST2 complex while the level of inhibitor of NF-κB α subunit (IκBα) in the NF-κB pathway was reduced, supporting that IL-33 activates both MAPK and NF-κB pathways (Figure 4A). Subsequently, we treated the HMC-1 cells with IL-33 C208S/C232S alone or pre-incubated with C2_2E12 and analyzed phosphorylation levels of ERK, JNK, and IκBα (Figure 4B,C). The relative phosphorylation level of IκBα is decreased by C2_2E12 in a dose-dependent manner, indicating the suppression of the NF-κB signaling pathway. Relative phosphorylation levels of ERK and JNK were reduced by C2_2E12 in a dose-dependent manner, implicating the suppression of the MAPK pathway. Analysis of the results indicated that C2_2E12 can neutralize IL-33 and ST2 interaction by binding with IL-33 in a dose-dependent manner (Figure 4B,C). Taken together, our results demonstrate that C2_2E12 treatment can reduce IL33/ST2 complex formation by interfering with IL-33 and ST2 binding and thus act as a neutralizing antibody for the suppression of the IL-33/ST2 signaling axis in cells.

## 3. Discussion

IL-33 has been associated with several chronic diseases such as asthma, atopic dermatitis, and inflammatory allergy, and recently, it was discovered that it plays important roles in regulatory immune responses. Neutralizing antibodies against IL-33 or ST2 have been developed to hinder IL-33 and ST2 binding. In this study, we discovered an scFv that specifically binds to IL-33 and subsequently interferes with IL-33 and ST2 complex formation. The IL-33 epitope region with C2_2E12 overlaps the ST2 binding domain in IL-33, implicating that C2_2E12 can act as a neutralizing antibody by competitive binding to IL-33. Pull-down assay and human cell line analysis verified that C2_2E12 has neutralizing efficacy. IL-33 and ST2 interaction stimulates the activation of immune cells such as mast cells, T-helper type 2 cells, and dendritic cells, thereby causing allergic inflammatory responses. Therefore, future evaluation of C2_2E12 and its refined clone(s) would desirably include the determination of efficacies in the keratinocyte/dendritic cells or epithelium/dendritic cell co-cultures. IL-33 is also known to stimulate type 2 innate lymphoid cells to release cytokines such as IL-5 and IL-13 [25]. Determination of the secreted IL-5 and IL-13 in response to the intervention of IL-33/ST-2 signaling axis by C2_2E12 would require future studies. C2_2E12 is not only a neutralizing antibody binding to IL-33, but also a monoclonal antibody discovered from human synthetic library of scFvs in vitro. The identification of epitope at the residue level, neutralizing the efficacy and human origin of C2_2E12 renders it a suitable candidate for further engineering. Our preliminary comparative data reveal that C2_2E12 shows an affinity only marginally inferior to that of a commercial antibody (data not shown). After further improving the affinity of C2_2E12 by affinity maturation and going through an in vivo test, C2_2E12 and its refined clone(s) could be used as a therapeutic antibody against IL-33 for treating allergic inflammatory diseases.

## 4. Materials and Methods

### 4.1. Plasmid Constructs Cloning

Genes encoding the mature form of IL-33 (residues 112–270), hereafter called simply “IL-33”, and the ectodomain of ST2 (residues 19–321) were synthesized (Cosmo Genetech, Seoul, Korea) and cloned into BamHI/StuI sites of parallel GST-2 vector [26] and *Not*I/*Nco*I sites of the pSF vector, respectively. CH2 and CH3 domains of human the IgG Fc region were cloned into XhoI/BsgI sites of pSF-ST2 plasmid for ST2-Fc fusion protein expression. Plasmids encoding IL-33 mutants (deletion mutants, alanine-scanning mutants, and an oxidation-resistant mutant C208S/C232S) were prepared by following the protocol for QuikChange kit (Agilent, Santa Clara, CA, USA). Identities of all the constructs were verified by DNA sequencing.

### 4.2. Expression and Purification of Recombinant Proteins

The plasmid encoding GST-IL-33 was transformed into *E. coli* BL21 (DE3) cells. A single colony was inoculated into 10 mL Luria broth (LB) media containing 100 μg·mL^−1^ ampicillin and grown at 37 °C overnight. After 16–18 h, the pre-cultured cells were transferred to 500 mL LB media containing 100 μg·mL^−1^ of ampicillin, grown at 37 °C until OD_600_ 0.6–1.0, induced with 0.6 mM isopropyl-β-d-1-thiogalactopyranoside (IPTG), and further grown at 25 °C overnight with gentle shaking. Cells were harvested by centrifugation and re-suspended in lysis buffer (50 mM Tris-HCl pH = 7.5 and 150 mM NaCl). Cells were disrupted by ultrasonication, cleared by centrifugation, and the supernatant containing GST-IL-33 was transferred to Glutathione Sepharose 4B resin (GE Healthcare, Chicago, IL, USA) pre-equilibrated with the lysis buffer. After washing the resin with the lysis buffer, GST-IL-33 was eluted in GST elution buffer (50 mM Tris-HCl pH 8.0, 150 mM NaCl, and 10 mM reduced glutathione). IL-33 was relieved from the GST fusion protein using a recombinant His_6_-tagged tobacco etch virus (TEV) protease during dialysis at 4 °C overnight. The resulting IL-33 was further purified on a Superdex 75 10/300 GL size-exclusion chromatography column (GE HealthCare, Chicago, IL, USA) pre-equilibrated with the lysis buffer. A ST2-Fc fusion protein, containing the ectodomain (residues 19–321) of ST2 and Fc from human IgG, was expressed in Expi293F cells (Thermo Fisher Scientific, Waltham, MA, USA) maintained in Expi293 expression medium (Thermo Fisher Scientific, Waltham, MA, USA). The day before transfection, cells were seeded to a final density of 2 × 10^6^ viable cells ml^−1^ in a 125 mL Erlenmeyer flask and grown at 37 °C for 24 h. After 24 h, cells were transfected with 30 μg of pSF-ST2-Fc plasmid DNA diluted in Opti-MEM™ I Medium (Thermo Fisher Scientific, Waltham, MA, USA) supplemented with 80 μL of ExpiFectamine™ 293 Reagent (Thermo Fisher Scientific, Waltham, MA, USA). Four days post-transfection, the cells were harvested by centrifugation, and its supernatant containing ST2-Fc was transferred to protein A agarose (Thermo Fisher Scientific, Waltham, MA, USA) pre-equilibrated by phosphate-buffered saline (PBS). The resin was washed using PBS, and ST2-Fc was eluted in Fc elution buffer (100 mM glycine pH = 3 with 1/10 volume of 1 M Tris-HCl pH = 8.0). The eluted ST2-Fc was dialyzed in the lysis buffer at 4 °C overnight.

### 4.3. Biopanning Using Phage Display

A synthetic human scFv library encoding His_6_- and HA-tagged scFv clones was used for biopanning [27]. Biopanning was performed as described previously with some modifications [28]. Library biopanning was performed in immuno tubes coated with the recombinant GST–IL-33 as an antigen in two conditions: inclusion of a negative selection with GST in every round (condition 1), and in the first round only (condition 2). In condition 1, five rounds of biopanning were performed in immuno tubes coated with GST protein for a negative selection and the recombinant GST-IL-33 at a gentle decrease in antigen concentrations (50, 10, 7.5, 5, and 2.5 μg·mL^−1^). In condition 2, the negative selection using GST protein is performed only in the first round of biopanning and coated with GST-IL-33 in the other four rounds of biopanning using the same concentration with condition 1. To select scFv clones that specifically bind to IL-33, single colonies from the final round of a biopanning output plate were grown in a 96-well cell culture plate until OD_600_ reached 0.6–1.0 and induced with 1 mM IPTG grown overnight at 30 °C with shaking. The harvested cells in each 96-well were re-suspended in cold 1× TES buffer (50 mM Tris-HCl pH = 8.0, 1 mM ethylenediaminetetraacetic acid (EDTA) and 20% (*w*/*v*) sucrose) for 30 min on ice, and cold 0.2×TES buffer was added to the re-suspended cells for 1 h on ice. The recombinant GST-IL-33 protein at 10 μg·mL^−1^ in PBS was coated on a 96-well ELISA plate. ELISA assay for scFv screening with horseradish peroxidase (HRP) conjugated anti-HA secondary antibody (1:3000 dilution, Santa Cruz Biotechnology, Dallas, TX, USA) was performed with a final reading of signals recorded at OD_450_. The OD_450_ values with GST-IL-33 were divided by the OD_450_ value with GST, and the ratio of OD_450_ values was compared.

### 4.4. Expression and Purification of scFvs in E. coli

Cells were pre-cultured from the single colonies of scFv-expressing *E. coli* BL21 (DE3) at 37 °C overnight, transferred to 500 mL super broth (SB) media containing 100 μg·mL^−1^ ampicillin. Cells were grown at 37 °C until OD_600_ 0.5–0.8 and induced with 1 mM IPTG at 30 °C with vigorous shaking. After 16–18 h, cells were harvested by centrifugation and re-suspended in cold 1× TES buffer for 30 min on ice, and cold 0.2× TES buffer was added to the re-suspended cells for 1 h on ice. The re-suspended cells supplemented with 5 mM MgCl_2_ to block EDTA were centrifuged, and their supernatants containing each scFvs were transferred to Ni-NTA agarose resin (Qiagen, Hilden, Germany). Each resin was washed by wash buffer A (PBS supplanted with 20 mM imidazole and 0.5 mM DTT) and scFvs were eluted by His-tag elution buffer A (PBS supplemented with 300 mM imidazole and 0.5 mM DTT). Size exclusion chromatography was performed on a Superdex 75 increase 10/300 GL column (GE HealthCare, Chicago, IL, USA) pre-equilibrated with PBS.

### 4.5. Reformatting, Expression, and Purification of C2_2E12 in Mammalian Cells

The gene encoding C2_2E12 in the pComb3X vector [27] was cloned into the pSF vector to express three different formats of C2_2E12 in mammalian cells: scFv, antigen-binding fragment (Fab), and immunoglobulin G (IgG). The C2_2E12 scFv was cloned into NotI/XhoI sites of the pSF vector for expression in Expi293F cells (Thermo Fisher Scientific, Waltham, MA, USA) maintained in Expi293 expression medium (Thermo Fisher Scientific, Waltham, MA, USA). A variable heavy chain and variable light chain of C2_2E12 were cloned into NotI/NcoI sites and HindIII/XhoI sites of the pSF vector, respectively, for the expression of Fab and IgG formats in Expi293F cells (Thermo Fisher Scientific, Waltham, MA, USA). Transfection and preparation steps for Fab and IgG were the same with those for scFv except for the amount and ratio of DNA plasmid used for transfection. The expression of scFv, Fab, and IgG formats of C2_2E12 in mammalian cells was performed in the same way as for ST2-Fc. Supernatants containing C2_2E12 scFv and Fab were transferred to Ni-NTA agarose resin (Qiagen, Hilden, Germany) pre-equilibrated with PBS. Each resin was washed by wash buffer B (PBS supplemented with 20 mM imidazole) and eluted by His-tag elution buffer B (PBS supplemented with 300 mM imidazole). Supernatant containing C2_2E12 IgG was transferred to a protein A agarose (Thermo Fisher Scientific, Waltham, MA, USA) pre-equilibrated by PBS. The resin was washed using PBS, and the C2_2E12 IgG was eluted in the Fc elution buffer. The antibodies were dialyzed at 4 °C overnight, concentrated, and loaded to a Superdex 75 10/300 GL size-exclusion chromatography column (GE HealthCare, Chicago, IL, USA) pre-equilibrated with PBS.

### 4.6. Immunoblot

Purified proteins were subjected to 12% SDS-PAGE and transferred onto polyvinylidene difluoride (PVDF) membranes (Merck Millipore, Burlington, MA, USA). Membranes were blocked with 5% (*w*/*v*) skim milk in Tris-buffered saline (pH = 7.5) containing 0.1% (*v*/*v*) tween-20 for 1 h at room temperature. Different primary antibodies were incubated at 4 °C overnight, and secondary antibodies were incubated at room temperature for 1 h. The bound antibody was detected by enhanced chemiluminescence (ECL) reaction with EZ-Western Lumi Pico kit (DoGen, Seoul, Korea).

### 4.7. Enzyme-Linked Immunosorbent Assay (ELISA)

GST-IL-33 was coated on half the total area of a Costar^®^ 96-well plate in the clear flat bottom polystyrene high bind microplate (Corning, Corning, NY, USA) and incubated at 4 °C for overnight. In the next day, the resulting culture was washed using PBST (PBS supplemented with 0.1% (*v*/*v*) tween-20) and blocked using blocking buffer (5% (*w*/*v*) skim milk in PBST). Serial dilutions of purified scFvs as the primary antibody with approximately 8 ng·mL^−1^ to 0.8 mg·mL^−1^ were added to the wells. The plate was incubated at ambient temperature for 1 h and washed using PBST. Subsequently, HRP-conjugated anti-HA antibody as the secondary antibody (1:3000 dilution, Santa Cruz Biotechnology) was added to the wells and incubated at ambient temperature for 1 h. The incubated plate was washed using PBST, and tetramethylbenzidine (TMB) substrate solution (GenDEPOT, Katy, TX, USA) was added for color development. After incubation for 10 min, 1 M H_2_SO_4_ was added to the plate to stop the color development reaction. The final signal readings were recorded at 450 nm and plotted using Prism 5 (GraphPad, San Diego, CA, USA).

### 4.8. Biolayer Interferometry (BLI)

Binding kinetics was measured by BLI experiments using a BLItz system (ForteBio, Fremont, CA, USA). *E. coli* cell lysate containing His_6_-tagged antibodies was prepared in 0.5× TES buffer. GST-IL-33 was prepared in BLI buffer (PBS supplemented with 20 mM imidazole, 0.05% (*v*/*v*) Triton X-100 and 0.1 mg·mL^−1^ BSA) to reduce the nonspecific binding signal. The BLI buffer was also used as the kinetics buffer. The cell lysate was immobilized to Ni-NTA biosensors (ForteBio, Fremont, CA, USA) and washed using the kinetics buffer. The sensors were subsequently reacted with various concentrations (2, 1, 0.5, and 0.25 μM) of GST-IL33 (association step) and washed using the kinetics buffer (dissociation step). These assays were performed twice each. All real-time recorded sensograms were analyzed by the ‘global fitting’ method in BLItz Pro 1.2 (ForteBio, Fremont, CA, USA) to calculate *k_on_* (association rate constant) and *k_off_* (dissociation rate constant) values. The *K*_d_ (dissociation constant) value of each antibody was calculated using the following equation:

(1) $$K_{d}=k_{off}/k_{on}.$$

*r*^2^ analysis, an indication of goodness of graph curve fitting, was performed using BLItz Pro 1.2, and the *r*^2^ values of all experiments were above 0.98. The graphs of raw sensograms were prepared by Prism 5 (GraphPad, San Diego, CA, USA).

### 4.9. Structural Modeling

A homology structural model for C2_2E12 that was generated using the SWISS-MODEL server [24] and the crystal structure of IL-33:ST2 complex (PDB ID: 4KC3) were used as templates for the docking of C2_2E12 to IL-33. Protein–protein docking modeling was performed using the HADDOCK server [29]. To perform the HADDOCK modeling, a restraint was applied such that the two key epitope residues of IL-33, L150 and K151, must interact with the complementary determination region of C2_2E12. The Z-score of clustering and other modeling parameters are listed in Table S2. Structural analysis of the interface between C2_2E12 and IL-33 was performed using PyMOL 1.8 (Schrödinger, New York, NY, USA).

### 4.10. Pull-Down Assay

Pull-down assay was performed using Glutathione Sepharose 4B resin (GE Healthcare, Chicago, IL, USA). First, 4 µM of GST-IL-33 was added to the resin and incubated for 30 min at 4 °C with gentle shaking. The resin was washed 4 times with wash buffer C (50 mM Tris-HCl pH = 7.5 and 150 mM NaCl) and incubated for 30 min at 4 °C with gentle shaking upon the addition of 4 µM of ST2-Fc. Subsequently, C2_2E12 at a series of concentrations (0.4, 4, 40, and 400 µM) was added to the resulting resin with further incubation for 30 min at 4 °C. After 4 times of washing, proteins were separated by SDS-PAGE on a 12% gel, stained with Coomassie Brilliant Blue, or transferred onto a PVDF membrane (45 mA for 60 min). Protein bands on the PVDF membrane were visualized by immunoblotting by ECL reaction using anti-ST2 (1:5000 dilution, Abcam, Cambridge, United Kingdom) and mouse anti-rabbit IgG–HRP (1:10000 dilution, Santa Cruz Biotechnology, Dallas, TX, USA), anti-GST-HRP (1:10000 dilution, Santa Cruz Biotechnology, Dallas, TX, USA), and anti-His_6_–HRP (1:10000 dilution, Santa Cruz Biotechnology, Dallas, TX, USA). Gels were quantified using ImageJ (National Institutes of Health, Bethesda, MD, USA).

### 4.11. Cell Signaling Analysis

HMC-1 cells were a kind gift from Prof. Soohyun Kim, Konkuk University, South Korea. HMC-1 cells were cultured in Iscove’s modified Dulbecco’s medium (IMDM) containing 10% FBS. HeLa cells were cultured in Dulbecco′s modified Eagle′s media (DMEM) containing 10% FBS. Cells were centrifuged at 800× *g* for 5 min at 4°C, and they were lysed with NETN lysis buffer (100 mM NaCl, 20 mM Tris-HCl pH = 8.0, 0.5 mM EDTA, 0.1% NP-40, 5 mM NEM, 10 mM NaF, 1 mM Na_3_VO_4_, a protein inhibitor cocktail) for 15 min on ice. Cell lysates were centrifuged at 15,000× *g* at 4°C for 10 min. Protein extracts were separated by SDS-PAGE and transferred onto PVDF membranes. Membranes were immunoblotted with the indicated antibodies, and the signals were visualized with ImageQuant™ LAS 4000 mini (GE Healthcare, Chicago, IL, USA). Anti-p-IĸB (#2859), anti-IĸB (#4814), anti-p-S6K (#9251), and anti-S6K (#9252) antibodies were purchased from Cell signaling. Anti-p-ERK (sc-7383), anti-ERK (sc-27129), and anti-IL-1RAcP (sc-376872) were purchased from Santa Cruz Biotechnology (Dallas, TX, USA). Anti-ST2 (#D065-3) were purchased from MBL (Woburn, MA, USA).

## 5. Patent

A patent application has been filed in South Korea (application number: 10-2020-0103135).

## Supplementary Materials

Supplementary materials can be found at https://www.mdpi.com/1422-0067/21/18/6953/s1.

## Abbreviations

IL-33	Interleukin-33
ST2	ST2, suppressor of tumorigenicity 2
scFv GST Fc	Single-chain antibody variable fragment glutathione *S*-transferase Antibody fragment crystallizable
