# Supplementary material for: Blocking of the IL-33/ST2 Signaling Axis by a Single-Chain Antibody Variable Fragment (scFv) Specific to IL-33 with a Defined Epitope
Source: Int J Mol Sci. 2020 Sep 22;21(18):6953. doi: 10.3390/ijms21186953 (PMC7554688; doi:10.3390/ijms21186953)
Supplement: Supplementary file 1 [file ijms-21-06953-s001.pdf]

International Journal of
Molecular Sciences

MDPI

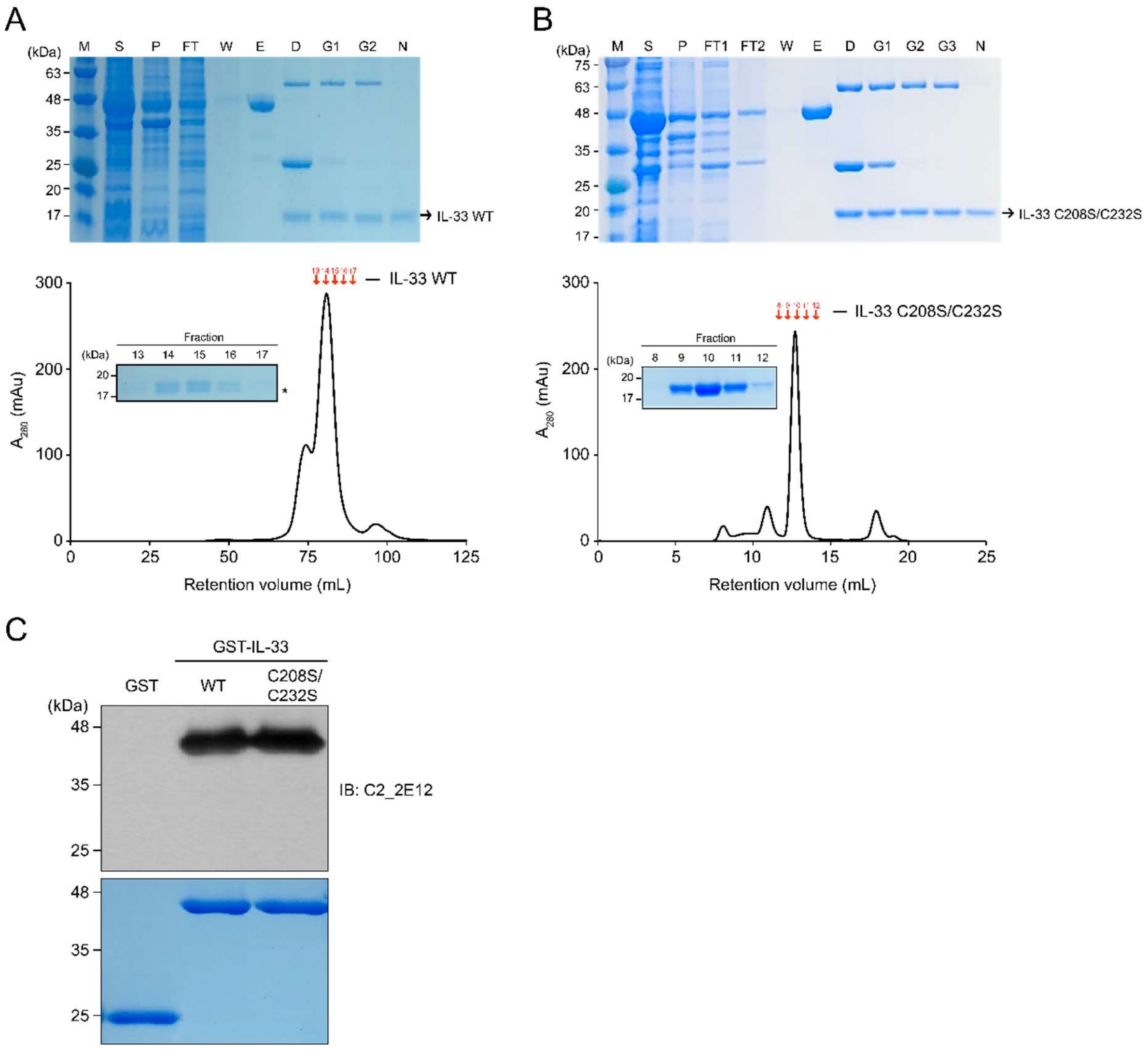

Supplementary Figure S1. Purification and characterization of IL-33 and C208S/C232S mutant. (**A**) (*Upper*) SDS-PAGE analysis of purification of GST-IL-33 wild type (WT). Lane M, protein size marker; lane S, supernatant; lane P, cell pellet; lane FT, flow-through after glutathione agarose binding; lane W, wash; lane E, elution of GST-IL-33 protein; lane D, GST-IL-33 digested with TEV protease during dialysis step; lanes G1 and G2, glutathione agarose binding 1-2 times to separate GST tag from IL-33; and lane N, Ni-NTA agarose binding to separate TEV protease from IL-33. (*Lower*) Elution chromatogram and SDS-PAGE analysis of analytic size exclusion chromatography of IL-33 WT. Lanes 13–17, fractions 13–17 on a HiLoad Superdex 75 pg 16/600 column. Fractions are marked with red arrow in the chromatogram. SDS-PAGE analysis of the fractions is shown. The oxidized form of IL-33 is indicated by asterisk (*). (**B**) (*Upper*) SDS-PAGE analysis of purification of GST-IL-33 C208S/C232S mutant. Lanes M through P, the same as those for IL-33 WT; lane FT1, the first flow-through after glutathione agarose binding; lane FT2, the second flow-through after glutathione agarose binding; lanes W through G2, the same as those for IL-33 WT; lane G3, the third time glutathione agarose binding to separate GST tag from IL-33 C208S/C232S; and lane N, Ni-NTA agarose binding to separate TEV protease from IL-33 C208S/C232S. (*Lower*) Elution chromatogram and SDS-PAGE analysis of analytic size exclusion chromatography of IL-33 C208S/C232S. Lanes 7–13: fractions 7–13 on a Superdex 75 increase 10/300 GL column. Fractions are marked with red arrow in the chromatogram. SDS-PAGE analysis of the fractions is shown. (**C**) Binding capacity of IL-33 WT

and C208S/C232S mutant with a selected scFv (C2_2E12) visualized by immunoblot analysis and SDS-PAGE. C2_2E12 was used as the primary antibody (0.5 $mg \cdot mL^{-1}$, 1:100 dilution) specific to IL-33 and anti-HA-HRP was used as the secondary antibody (0.2 $mg \cdot mL^{-11}$, 1:5000 dilution).

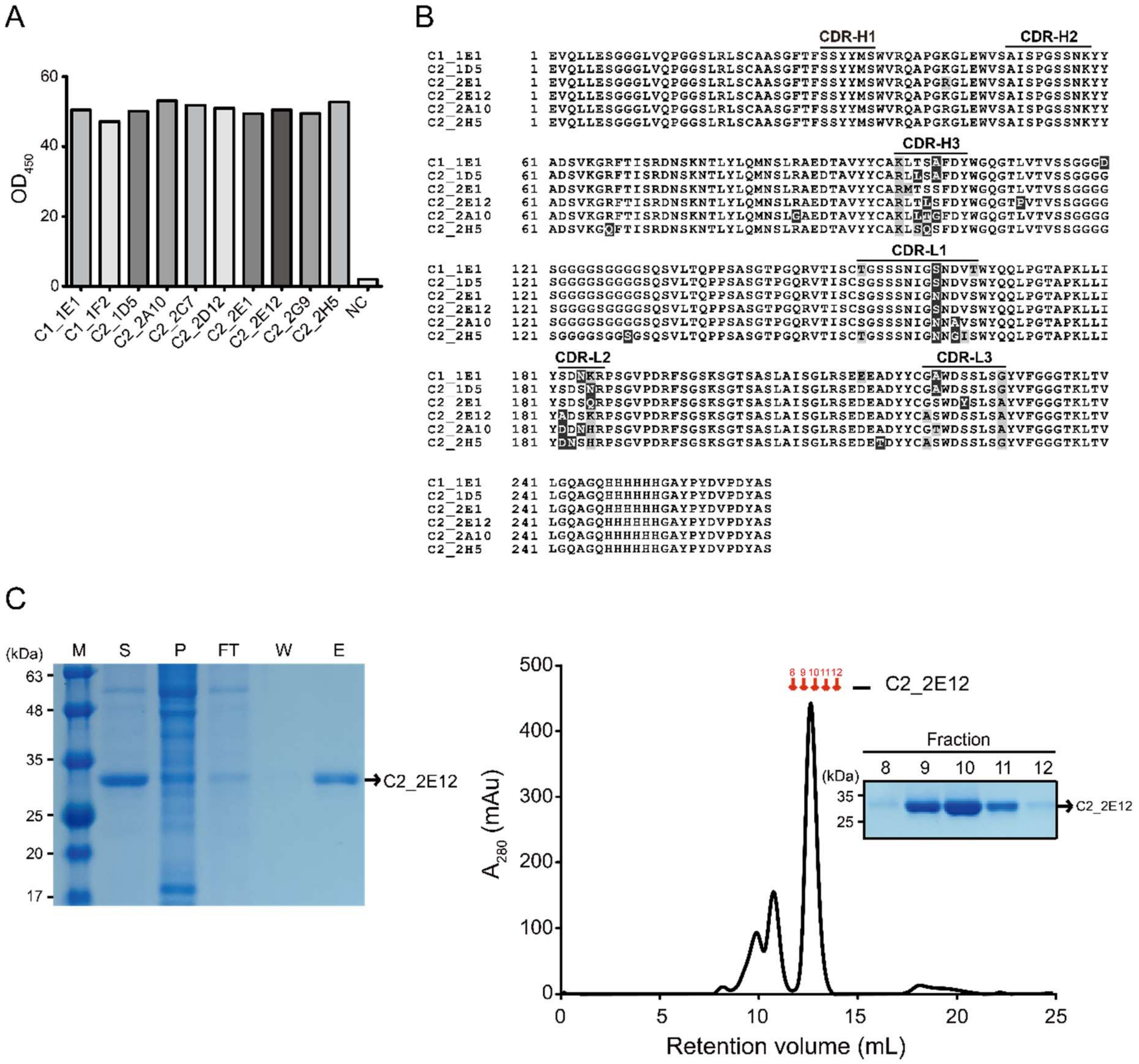

Supplementary Figure S2. Biopanning of scFv clones specific to IL-33. (**A**) OD_{450} ratio of the top 10 clones out of 96 clones that exhibited high binding signals and the negative control (labeled NC) from the final round of bio-panning by ELISA. Each well of ELISA plates was coated with recombinant GST-IL-33 and GST as antigen. 384 colonies from final 5th round of panning were subject to ELISA analysis. Among the 384 clones, top 10 clones were selected by comparing the OD_{450} values. (**B**) Amino acids sequences of the six scFvs (C1_1E1, C2_1D5, C2_2A10, C2_2E1, C2_2E12 and C2_2H5) selected from bio-panning are shown in one letter codes. Six CDR regions of variable heavy chain and variable light chain are labeled. (**C**) (*Left*) SDS-PAGE analysis of recombinant C2_2E12 scFv antibody purification (lane M, protein size marker; lane S, antibody secreted media; lane P, cell pellet; lane FT, flow-through after Ni-NTA agarose binding; lane W, wash; and lane E, elution of C2_2E12). (*Right*) Elution fraction graph and SDS-PAGE analysis after analytic size exclusion chromatography of C2_2E12 (lanes 8–12: eluted fractions 8–12) on a Superdex 75 increase 10/300 GL column. Fractions are marked with red arrow in the graph. SDS-PAGE analysis of the fractions is shown.

Supplementary Table S1. Library biopanning titers for anti-IL-33 scFv screening

Condition 1

Round	Antigen concentration ($\mu g \cdot ml^{-1}$)	Titer of input (cfu[a])	Titier of ouput (1/10) (cfu[a])	Titer of ouput (1/100) (cfu[a])
1	50	1.6×10^{12}	2.9×10^{8}	3.0×10^{8}
2	10	1.9×10^{11}	1.6×10^{7}	3.6×10^{7}
3	7.5	1.2×10^{13}	8.8×10^{7}	8.0×10^{7}
4	5	1.0×10^{11}	5.4×10^{6}	1.1×10^{7}
5	2.5	1.9×10^{11}	1.5×10^{8}	1.2×10^{8}

[a]cfu, colony forming unit.

Condition 2

Round	Antigen concentration ($\mu g \cdot ml^{-1}$)	Titer of input (cfu[a])	Titier of ouput (1/10) (cfu[a])	Titer of ouput (1/100) (cfu[a])
1	50	1.6×10^{12}	2.9×10^{8}	3.0×10^{8}
2	10	1.7×10^{11}	3.7×10^{7}	3.0×10^{7}
3	7.5	1.0×10^{13}	6.8×10^{7}	1.8×10^{7}
4	5	9.0×10^{10}	3.4×10^{7}	2.5×10^{7}
5	2.5	7.0×10^{10}	3.4×10^{7}	2.3×10^{8}

Supplementary Table S2. HADDOCK summary for the docking of C2_2E12 with IL-33

Molecule	C2_2E12
HADDOCK score (A.U.[a])	-97.8 ± 11.4
Cluster size	5
R.m.s.d. from the overall lowest-energy structure (Å)	0.5 ± 0.3
van der Waals energy ($kcal \cdot mol^{-1}$)	-40.0 ± 2.9
Electrostatic energy ($kcal \cdot mol^{-1}$)	-261.1 ± 60.1
Desolvation energy ($kcal \cdot mol^{-1}$)	-6.8 ± 5.6
Restraints violation energy ($kcal \cdot mol^{-1}$)	12.2 ± 14.47
Buried surface area ($Å^2$)	1350.8 ± 90.0
Z-score	-1.3

[a]A.U., arbitrary unit.
